# Reproductive health awareness among adolescents in Kazakhstan: a pre-post quasi- experimental study

**DOI:** 10.3389/frph.2026.1770464

**Published:** 2026-03-19

**Authors:** Saule Derbisbek, Aigul Abduldayeva, Zaituna Khamidullina, Gulnur Doszhanova, Nurikamal Kurmanbek, Aigerim Ospanova

**Affiliations:** 1 Department of Research Institute of Preventive Medicine Named Academician E.Dalenov and Department of Obstetrics and Gynecology, Astana Medical University, Astana, Kazakhstan; 2 Department of Obstetrics and Gynecology, Multidisciplinary City Hospital, Astana, Kazakhstan

**Keywords:** adolescent, contraception knowledge, health awareness, reproductive health, sexual health education

## Abstract

**Background:**

Adolescence is a critical developmental period marked by significant physical, psychological, and social changes, making reproductive health literacy essential for ensuring adolescents’ well-being.

**Methods:**

A questionnaire-based pre-post quasi-experimental study conducted among adolescents aged 15–19 years in Astana during the 2024–2025 academic year. The questionnaire consists of 17-item assessing knowledge of contraception, prevention of sexually transmitted infections, puberty, and menstrual health. The same participants completed the questionnaire both before and after a single 90- minute educational intervention session, allowing for paired comparisons of knowledge gains.

**Results:**

A total of 180 students recruited from public and private schools in the capital City of Kazakhstan, Astana. The results showed significant gaps in reproductive health knowledge, with 53.3% of participants correctly identified contraceptive methods, and fewer than half demonstrated accurate understanding of STI prevention. Knowledge of puberty, secondary sexual characteristics, and menstrual health was similarly limited, with many students reporting anxieties or misconceptions. Most adolescents relied on the internet (52.2%) as their primary source of information, whereas parents and schools contributed minimally (both 23.3%). Despite these gaps, understanding of sexual consent was relatively high (90%). The educational intervention appeared to improve the overall reproductive health awareness, including STI prevention and recognition of normal developmental changes.

**Conclusion:**

Adolescents in Kazakhstan exhibited poor knowledge about reproductive health. This could be due to sociocultural norms and limited formal reproductive health education. The marked improvement following a brief educational session demonstrates the effectiveness of targeted interventions and highlights the importance of developing culturally tailored strategies to strengthen adolescent reproductive health literacy.

## Introduction

Adolescence is a critical transitional period between late childhood and early adulthood, characterized by profound biological, psychological, and social transformations (1). This developmental stage involves not only sexual maturation through hormonal and physical changes, but also increased complexity in group interactions and social behaviors (2).

One of the important health aspects during this period is reproductive health, a state of complete physical, mental, and social well-being in all matters related to the reproductive system and its functions, encompassing physical, emotional, behavioral, and social dimensions (3, 4). Lack of attention to adolescent's reproductive health contributes to significant health burdens worldwide, as adolescents often exposed to numerous vulnerabilities including; unintended pregnancies, unsafe abortions, sexually transmitted infections (STIs) such as HIV, as well as any form of sexual coercion or violence (5).

For example, the UNICEF-adolescent HIV prevention reported that in the year 2024 alone approximately 370,000 young people between (between15–24 years old) were newly infected with HIV, including about 145,000 adolescents aged 15–19 years old, with girls disproportionately affected, making up nearly 71% of all cases in this age group (6). In addition to STIs, and despite global improvements in maternal health, adolescent pregnancy continues to pose a substantial public health challenge, particularly in low- and middle-income countries, where roughly 19% of young women become pregnant before turning 18 (7).

Kazakhstan, a developing middle-income country in Central Asian, has a population of 20.3 million, including 3.5 million adolescents aged 10–19 years (17.2%), of whom 231,710 adolescents residing in the capital city, Astana (8). Oftentimes, adolescents in Kazakhstan face significant challenges related to their reproductive health education, with multiple systemic barriers hindering access to accurate, age- appropriate information, including the sociocultural context, which creates substantial obstacles to open dialogue about sexual and reproductive health (9). Insufficient comprehensive sexuality education and prevailing cultural expectations restrict open communication about adolescents’ reproductive and sexual health between parents and young people, resulting in misinformation and poor access to credible sources (10). Therefore, effective interventions must consider the sociodemographic determinants that shape adolescents’ knowledge, attitudes, and practices.

Consequently, overcoming these constraints calls for establishing a solid base of accurate information, encouraging supportive attitudes, and promoting healthy practices, ultimately advancing outcomes and strengthening adolescents’ overall health and well-being.

However, understanding the baseline knowledge and identifying specific gaps are essential prerequisites for developing culturally appropriate, evidence-based educational interventions. Therefore, there is a need to investigate the current state of reproductive health awareness among Kazakhstan adolescents to inform policy development, curriculum design, and targeted intervention strategies.

Therefore, this study aims to evaluate adolescents’ understanding of contraception, STI prevention, pubertal changes, and menstrual health among adolescents aged 15–19 years in Astana, as well as to determine the primary sources from which they obtain reproductive health information. In addition, the study will examine their attitudes toward sexual health education and communication patterns regarding reproductive health topics with parents, educators, and healthcare professionals.

We hypothesized that by addressing these objectives, we will be able to provide evidence-based recommendations for developing comprehensive reproductive health education programs tailored to the needs of adolescents in Kazakhstan.

## Methods

### Ethics approval

The research project was submitted and approved by the Local Bioethics Committee of Astana Medical University (Protocol No. 12, September 2024)—Kazakhstan. Permissions to conduct the study obtained from the heads of participating schools and universities, and written informed consents obtained from all participants after they were informed about the study's purpose, potential risks and benefits, and that their participation is voluntary and they have the right to withdraw at any time. For participants under 18 years of age, parental or legal guardians consented to participate in this study.

### Study setting and design

This is a questionnaire-based pre-post quasi-experimental study that was conducted in Astana, the capital city of the country with both Kazakh and Russian ethnic groups. The study conducted during the 2024 -2025 academic year, approximately between September-July. A 17-item multiple-choice questionnaire with good reliability and internal consistency (Cronbach's alpha coefficient of 0.82), divided into different sections; including a section about demographic data and sections designed to test reproductive health knowledge among students in Astana. Each item was coded as “1” for a correct response and “0” for an incorrect or “don’t know” response. A composite reproductive health awareness score was calculated by summing the number of correct responses, yielding a total possible score ranging from 0 to 17, with higher scores indicating greater awareness. For ease of interpretation, total scores were also converted to percentages (score/17 × 100). Change scores were calculated as the difference between post-intervention and pre-intervention total scores for each participant (Post/Pre).

A single 90-minute educational intervention was conducted, covering puberty, sexual health, contraception, sexually transmitted infections, and healthy relationships through interactive lectures, group discussions, and multimedia presentations. The session was delivered by trained educators, with support from medical doctors and psychologists, using standardized materials designed for the study.

The content was tailored to the age and gender of the participants to ensure relevance and comprehension, as well as enhancing transparency and facilitating reproducibility of the intervention. Participants included both male and female students aged 15–19 years. Students were surveyed before and immediately after the intervention via face-to-face interviews using the questionnaire, and to ensure confidentiality, the interviews conducted privately. The questionnaire was piloted with 25 participants (16 young adolescents aged 15–17 and 9 older adolescents aged 18–19) to assess clarity, readability, and comprehensibility of the items. Minor revisions were made based on participant feedback, and the finalized version was reviewed by subject-matter experts to ensure content validity before being used in the main study.

### Study population (inclusion/exclusion)

Participants recruited by convenience sampling method from four different secondary schools in Astana region including students from one private and three public secondary schools in grades 7–11, and first-year university students, some of whom were from other regions of Kazakhstan.

Participants were not asked for their names, email address or contact information, ensuring the privacy of survey respondents. Consenting students within the specified age group who attended the educational intervention were eligible for inclusion. Students younger than 15 or older than 19 years old, those who did not attend the intervention session, and did not submit their questionnaires or questionnaires with missing responses were excluded from the study. Completed questionnaires were assigned unique codes to maintain anonymity while allowing for data tracking. All forms were checked for completeness by trained researchers, and the data were entered into Microsoft Excel for analysis.

### Sample size and power calculation

Sample size calculations were performed *a priori* using G*Power software (version 3.1.9.7, Heinrich- Heine-Universität Düsseldorf, Germany). Given that the primary objective of the study was to evaluate change in awareness following the educational intervention, the sample size calculation was based on a paired t-test (matched-pairs design), consistent with the pre/post study design. This ensures that the power analysis directly reflects the main outcome of interest, namely the subject mean difference in awareness scores before and after the intervention (29, 30).

The study measured knowledge levels in a single cohort of adolescents (both boys and girls) at two time points; before and after an educational intervention. Accordingly, the paired t-test framework was selected for both the primary analysis and the *a priori* power calculation to ensure conceptual and statistical consistency between study design, hypothesis, and analysis.

Furthermore, an effect size of Cohen's d = 0.5 was chosen, reflecting a medium effect size according to Cohen's framework (11). For the paired design, this corresponds to a standardized mean difference in pre–post scores (Cohen's dz) of 0.5, representing a moderate and practically meaningful improvement in awareness following the intervention. This represents a difference that is both educationally and clinically meaningful, based on public health considerations, rather than being driven solely by statistical detectability. Prioritizing effect size ensures that the study emphasizes practical significance in adolescent reproductive health knowledge, rather than trivial differences that may achieve statistical significance without educational relevance (12). A medium effect size of 0.5 corresponds to a knowledge gap substantial enough to justify targeted intervention.

Power analysis parameters included a two-tailed test (to detect whether awareness scores were higher or lower than the threshold), *α* = 0.05, d = 0.5, and powe*r* = 0.95. This yielded a minimum required sample size of 54 participants. The study deliberately used a conservative approach with higher power than the conventional 0.80 to reduce the risk of underpowering while ensuring detection of meaningful within-subject knowledge differences.

### Data storage

All data collection forms were securely stored in a locked location to ensure confidentiality and data integrity. Access was strictly limited to the Principal Investigator, who was responsible for maintaining the security of the information and overseeing proper data handling throughout the study.

### Statistical analysis

Statistical analyses performed using IBM SPSS Statistics version (26.0). The normality of continuous variables assessed using the Kolmogorov–Smirnov test. Normally distributed continuous variables presented as mean ± standard deviation (M ± SD), while non-normally distributed variables reported as median with interquartile range [Me (Q1–Q3)]. Descriptive statistics, including frequency distributions and percentages were calculated to characterize respondents’ socio-demographic characteristics (age, gender, grade level, school type), paired t-tests to compare reproductive health knowledge scores pre and post reproductive health intervention session.

## Results

The final recruited sample included 180 participants, far exceeding the minimum requirement and resulting in achieved power >0.99. Notably, the sample size was capped at 180 participants to balance feasibility and resources while ensuring adequate statistical power and generalizability. However, this larger sample provides several advantages: (1) strong ability to detect meaningful differences in knowledge, (2) sufficient power for subgroup analyses (e.g., by gender, age, and school type), (3) buffer against missing or incomplete data, and (4) improved precision in estimating mean awareness scores with narrow confidence intervals. This substantial oversampling ensures that conclusions about adolescent reproductive health awareness are robust, reliable, and minimally affected by sampling variability.

### Demographic data

The study included 180 adolescent students with an equal gender distribution, comprising 50% females

and 50% males. The participants’ mean age was 16.06 years (SD = 0.876), reflecting a relatively narrow age range. Most participants were enrolled in public schools (83.4%), while the remaining attended private institutions, suggesting that the sample predominantly reflects the experiences of students in government- run educational settings. In terms of residential background, a majority of the adolescents (67.7%) resided in urban areas, while the remainder came from suburban or rural regions, demonstrating potential urban vs. rural differences in access to educational resources and reproductive health information. Table 1 provides a detailed overview of the demographic characteristics of the study population.

**Table 1:** Profile of the respondents.

Variable	Group	Number	Percentage
Gender	Female	90	50%
	Male	90	50%
Age of the student (years)	15	53	29.4%
	16	75	41.6%
	17	41	22.7%
	18	11	6.1%
Mean age = 16,06; SD 0.876			
Management of school and University	Private government	30 150	16.6% 83.4%
Locality of the students	Rural	60	33.3%
	Urban	120	67.7%
Total		180	100%

### Reproductive health awareness among adolescents

The seventeen multiple-choice questions, each with a single correct response, were employed to assess reproductive health awareness among higher secondary school students. Participants’ responses were aggregated to calculate an overall reproductive health awareness score, providing a quantitative measure of their knowledge across key domains such as contraception, sexually transmitted infection prevention, puberty, and menstrual health. The distribution of scores and detailed results for each question are presented in Table 2.

**Table 2:** Knowledge of adolescent on reproductive and sexual health before and after educational session.

Characteristics	Frequency/Percentage of results before session	Frequency/Percentage of results after session
Knowledge about contraception?		
Methods to prevent pregnancy.	96 (53.3%)	180 (100%)
Exercise to improve physical fitness.	47 (26.1%)	
Tools to improve academic performance.	37 (20.6%)	
Knowledge about preventing sexually transmitted diseases (Select all that apply)		
Using condoms.	80 (44%)	175 (97.2%)
Washing your hands after being outside.	11 (6.1%)	0
Vaccination.	29 (16.1%)	0
Abstaining from sexual intercourse.	60 (33.3%)	5 (2.8%)
Do you think it is important to have knowledge about sex education?		
Yes, it's important for everyone.	110 (61.1%)	180 (100%)
I'm not sure.	70 (38.9%)	0
No, it's not that important.	0	0
Where do you get information about sex education? (Select all that apply)		
Internet.	94 (52.2%)	10 (5.6%)
School.	42 (23.3%)	20 (11.1%)
Friends.	0	0
Parents.	0	0
Healthcare professionals.	44 (24.4%)	150 (83.3%)
Do you feel informed enough to make decisions about your sexual life?		
Yes, I'm completely sure.	95 (52.8%)	171 (95%)
Somewhat sure.	50 (27.8%)	0
No, I consider it a lack of information.	35 (19.4%)	9 (5%)
What are secondary sexual characteristics?		
Characteristics that develop during puberty and changes not directly related to reproductive regression.	122 (67.8%)	158 (87.8%)
Primary sex characteristics (e.g., presence of ovaries or testes).	42 (23.3%)	17 (9.4%)
Genetic traits passed down from parents.	16 (8.9%)	5 (2.8%)
The first development of secondary sexual characteristics in boys begins with?		
Growth of facial and body hair.	52 (28.9%)	75 (41.7%)
Enlargement of the breasts.	0	0
Voice change.	73 (40.6%)	30 (16.7%)
Growth of the genitals.	32 (17.8%)	61 (33.9%)
Appearance of acne and acne fluid.	23 (12.8%)	14 (7.8%)
The first development of secondary sexual characteristics in girls begins with?		
Breast development.	39 (21.7%)	148 (82.2%)
Onset of menstruation.	55 (30.6%)	1 (0.6%)
Growth of underarm and pubic hair.	31 (17.2%)	3 (1.6%)
Change in body shape (more rounded hips).	55 (30.6%)	0
Increase in height.	0	0
Do you know what is normal and what may be cause for concern when secondary sexual characteristics develop?		
Yes, I understand the difference well.	51 (28.3%)	112 (62.2%)
I partially know, but sometimes I have doubts.	75 (41.7%)	68 (37.8%)
No, I don't understand.	54 (30%)	0
Where do you get information about secondary sexual characteristics?		
School (biology classes, life safety).	30 (16.7%)	30 (16.7%)
The Internet.	75 (41.7%)	27 (15%)
Friends.	25 (13.9%)	2 (1.1%)
Parents.	0	0
Medical professionals.	50 (27.8%)	121 (67.2%)
Do you feel confident knowing about the changes that are constantly happening in your body?		
Yes, I am completely sure.	53 (29.4%)	108 (60%)
Most of the time I am sure, but sometimes I have questions.	100 (55.6%)	71 (39.4%)
No, I think it's a lack of information.	27 (15%)	1 (0.55%)
Do you know how to properly care for yourself during puberty (for example, how to care for your skin, what hygiene products to use)?		
Yes, I'm well educated.	59 (32.8%)	131 (72.8%)
I know partly, but I want to learn more.	101 (56.1%)	49 (27.2%)
No, I don't understand it.	20 (11.1%)	0
How do you feel about the onset of menstruation? (For female only)		
I understand that it's a normal part of growing up.	33 (36.7%)	80 (88.9)
I'm a little worried, but I know it's normal.	47 (52.2%)	10 (11.1%)
It scares me, I don't know what to do.	10 (11.1%)	0

### Contraception knowledge

Awareness of contraception among adolescents was moderate. Only 53.3% (*n* = 96) correctly identified contraceptive methods, while nearly half (46.7%) held misconceptions. This indicates a considerable gap in basic knowledge of pregnancy prevention, highlighting the need for targeted interventions to support informed decision-making.

### STI prevention

Knowledge of STI prevention was limited. Fewer than half of participants (44%, *n* = 80) recognized condom use, and only 33.3% (*n* = 60) identified abstinence as an effective preventive measure. While 61.1% (*n* = 110) acknowledged the importance of sex education, only 52.8% (*n* = 95) felt confident making sexual health decisions, suggesting that awareness alone does not necessarily translate into self- efficacy.

### Sources of reproductive health information

The internet emerged as the predominant source of reproductive health information (52.2%, *n* = 94), whereas parental guidance (23.3%, *n* = 44) and school-based education (23.3%, *n* = 42) played a minimal role. These findings underscore adolescents’ reliance on digital platforms and the need to strengthen both familial and school-based education to provide accurate, trustworthy information. Interestingly, following the educational session, the proportion of adolescents identifying healthcare professionals as their primary source of information significantly increased, while reliance on the internet declined. However, this post-intervention change likely represents immediate exposure to the educational session rather than sustained changes in adolescents’ habitual information-seeking behavior.

### Knowledge of puberty and secondary sexual characteristics

Substantial gaps were observed in understanding puberty and secondary sexual characteristics. Only 67.8% (*n* = 122) correctly defined these characteristics, and fewer than one-third could identify the initial developmental changes in boys (28.9%, *n* = 52) or girls (21.7%, *n* = 39). Most participants (71.7%) reported partial or no understanding of normal vs. concerning developmental patterns, highlighting the necessity for comprehensive, structured education on pubertal development.

### Self-care and menstrual health

Knowledge of puberty-related self-care was inadequate, with only 32.8% (*n* = 59) reporting feeling well-informed about hygiene practices. Among female participants, 63.3% expressed anxiety or fear regarding menstruation, whereas only 36.7% (*n* = 33) viewed it as a normal physiological process. These findings emphasize the importance of integrating menstrual health education into broader reproductive health programs to address misconceptions and alleviate concerns.

### Understanding of sexual consent

The majority of participants (90%, *n* = 162) demonstrated a clear understanding of sexual consent, indicating a high level of awareness regarding personal autonomy and the importance of mutual agreement in sexual interactions (Table 3). This suggests that educational interventions or prior exposure to reproductive health information may have positively influenced adolescents’ comprehension of consent-related issues.

**Table 3:** Adolescent's behavior on reproductive and sexual health before and after educational session.

Characteristics of respondents	Results of respondents before session *n* (%)	Results of respondents after session *n* (%)
What is a sexual consent?		
When both partners voluntarily agree to intercourse	162 (90%)	175 (97.2%)
When one partner decides for both.	5 (2.8%)	5 (2.8%)
Not necessarily, if you're already in a relationship	13 (7.2%)	
How do you feel about discussing sexual literacy with friends or adults?		
Easily and freely	63 (35%)	63 (35%)
A little shy	60 (33.3%)	60 (33.3%)
I never discuss it, it's too uncomfortable	57 (31.7%)	57 (31.7%)
Have you ever used contraception?		
Yes.	52 (28.9%)	52 (28.9%)
No.	63 (35%)	63 (35%)
I prefer not to.	65 (36.1%)	65 (36.1%)
Do you think it's appropriate to discuss issues related to sexuality with your parents or other adults?		
Yes, it's important	63 (35%)	110 (61.1%)
Sometimes, depending on the situation	48 (26.7%)	70 (38.9%)
No, I prefer not to discuss these topics	69 (38.3%)	

### Communication barriers

Despite strong knowledge of consent, significant barriers to open discussion about sexual topics were evident. Approximately 65% of participants reported feeling shy or entirely unwilling to discuss sexual matters, highlighting persistent social and cultural constraints that limit adolescent communication on sensitive issues (Table 3). These findings show the need for safe, supportive environments that encourage dialogue about sexual and reproductive health.

### Contraceptive knowledge and parental communication

When asked about contraceptive use, 36.1% of students (*n* = 65) preferred not to respond, reflecting discomfort or uncertainty around the topic. Moreover, 38.3% (*n* = 69) expressed unwillingness to discuss sexuality with parents or other adults, emphasizing a gap in intergenerational communication regarding reproductive health. This suggests that while basic knowledge of consent is widespread, broader aspects of sexual health, particularly contraceptive practices and open dialogue with trusted adults, remain areas where adolescents require further guidance and support (Table 3).

### Impact of the educational intervention on reproductive health knowledge

A paired t-test was conducted to compare reproductive health awareness scores before and after the educational session. The results demonstrated a significant improvement in overall knowledge across most domains (*p* < 0.05), highlighting the effectiveness of the intervention. Significant gains in understanding STI prevention, general reproductive health awareness, recognition of secondary sexual characteristics, and the ability to distinguish between normal and abnormal body changes were observed. The only exception was knowledge related to pubertal changes in boys, which showed an upward trend, but did not reach statistical significance (*p* = 0.061) of 3.7 (95% CI:2.9–4.5; paired t-test, *p* < 0.001).These findings indicate that a single, interactive educational session can produce meaningful improvements in adolescent reproductive health knowledge, particularly in areas previously identified as knowledge gaps. The observed gains are not only statistically significant but also practically important, as enhanced understanding of reproductive health concepts is likely to support informed decision- making, promote healthy behaviors, and reduce misconceptions among adolescents (Table 4).

**Table 4:** Comparison via paired *t*-test of reproductive health awareness before and after educational session.

Variables before/after session	Mean ± SD	(95% CI: lower-upper)	*P*-value
How to prevent STI?	0.483 ± 0.881	(0.353 ± 0.613)	<0.001
What is a sexual consent?	0.144 ± 0.519	(0.068 ± 0.220)	<0.001
Where do you get information about sex education?	−2.789 ± 1.641	(−3.030 ± −2.548)	<0.001
Do you feel informed enough to make decisions about your sexual life?	0.587 ± 0.733	(0.480 ± 0.694)	<0.001
The first development of secondary sexual characteristics in girls begins with?	0.261 ± 0.563	(0.178 ± 0.344)	<0.001
The first development of secondary sexual characteristics in boys begins with?	0.194 ± 1.383	(−0.009 ± 0.397)	>0.061
Do you know what is normal and what may be cause for concern when secondary sexual characteristics develop?	0.639 ± 1.029	(0.488 ± 0.790)	<0.001
Where do you get information about secondary sexual characteristics?	−0.63 ± 1.546	(−0.857 ± −0.403)	<0.001
Do you feel confident knowing about the changes that are constantly happening in your body?	0.244 ± 0.622	(0.153 ± 0.335)	<0.001
Do you think it's appropriate to discuss issues related to sexuality with your parents or other adults?	0.644 ± 0.869	(0.516 ± 0.772)	<0.001
Do you know how to properly care for yourself during puberty (for example, how to care for your skin, what hygiene products to use)?	0.344 ± 0.477	(0.274 ± 0.414)	<0.001
How do you feel about the onset of menstruation? (For female only)	0.389 ± 0.489	(0.317 ± 0.461)	<0.001

## Discussion

The current study provides a comprehensive assessment of reproductive health awareness among adolescents in Astana, showing substantial knowledge gaps that have significant implications for public health policy and educational programming. Despite residing in an urban setting with relatively greater access to educational resources, adolescents demonstrated insufficient knowledge across multiple domains of reproductive health. The significant improvements in awareness observed following the educational session demonstrated both the poor understanding of the participants about reproductive health and the potential impact targeted interventions could have. These findings emphasize the urgent need for culturally sensitive, evidence-based comprehensive reproductive education programs in Kazakhstan.

A particularly concerning finding of this study is that only 53.3% of adolescents correctly identified contraception as a method for preventing pregnancy, with nearly half of participants holding significant misconceptions. This gap in knowledge is especially troubling given that adolescent pregnancy rates in Kazakhstan remain high, and insufficient contraceptive knowledge is a well-established risk factor for unintended pregnancies (13). Our results are consistent with previous research from Russia, Bashkortostan, Tatarstan, and other Central Asian regions, which reported similar inadequate contraceptive awareness among young people (14–16). Notably, the magnitude of such poor knowledge in an urban, relatively well-educated sample suggests that the situation may be even more pronounced in rural areas where access to educational resources is limited. Similarly, a study by Castro et al., that investigated knowledge and attitudes regarding contraceptive methods and sex education in students and parents of several schools across Colombia, showed that 52.3% only had adequate knowledge about contraceptives (17). These findings are also consistent with those from Lapeira and colleagues, who studied 64 adolescents in the Caribbean, and reported that 54.7% of respondents have an acceptable knowledge about contraceptive methods (18).

Interestingly, there is a significantly poor knowledge about STI prevention among participants, with only 44% correctly identifying condom use as an effective preventive measure. This finding is particularly concerning given the rising rates of STIs among adolescents worldwide (19, 20). Moreover, 6.1% of respondents erroneously believed that hand-washing could prevent STIs, reflecting not only a lack of knowledge, but also the presence of significant misconceptions that may foster a false sense of security and encourage risky sexual behaviors. These results are consistent with previous studies in both developed and developing contexts. For example, research among adolescents in Nigeria and India reported similarly low levels of accurate knowledge regarding condom use and STI prevention, alongside persistent misconceptions about ineffective preventive practices (21). The recurrence of such knowledge deficits across diverse settings highlights the urgent need for targeted, evidence-based sexual health education interventions that address both factual information and prevalent myths, thereby reducing risk behaviors and improving adolescent sexual health outcomes.

However, when asked about the source of sexual health information, the results show that 52.2% of adolescents rely primarily on the internet for sexual health information, whereas only 23.3% turn to parents or schools, underscores a growing shift toward digital sources. This pattern aligns with international data. For example, in a systematic review of 48 studies by Silva et al., reported the internet is among the most common sources of sexual information for adolescents, especially when anonymity and perceived reliability are important (22).

Similarly, a cross-sectional study by Rosen et al. that investigated students’ preferences when looking for sexual health information, reported that nearly all surveyed adolescents value easily accessible, understandable online sexual-health resources (23). In the context of Kazakhstan and Central Asia, cultural taboos around discussing sexuality have long limited the role of traditional education and parental guidance. According to journalism and policy commentary, sexuality education in Kazakhstani schools is not systematically implemented, which constrains reliable in-person sources of information (24). In a qualitative study by Kabatova titled “Overcoming a Taboo: Normalizing Sexuality Education in Kazakhstan” highlighted how, in the absence of formal sex education, some Kazakh youth turn to informal online spaces to learn and talk about sexual and reproductive health (9). In low-resource or culturally conservative settings such as in Kazakhstan, the internet's role can become even more pronounced, as many adolescents, particularly, girls use the Internet for reproductive health knowledge due to privacy, freedom of access, and a lack of alternatives in formal education or family discussion (25).

While the internet offers clear benefits, such as convenience, anonymity, and wide access, it also poses risks, as adolescents may encounter misinformation, incomplete content, or content that normalizes risky behavior, particularly if they lack digital health literacy. Such a discrepancy between convenience and correctness suggests that exclusive dependence on digital platforms, without strengthening traditional sources of sexual health education, might be harmful.

Interestingly, the results showed no significant difference in the mean scores of knowledge andattitudes between males and females (*p* = 0.386), with both genders demonstrating similar mean awareness scores (males: 8.45, SD = 2.34; females: 8.72, SD = 2.51 out of 17 possible points). This finding is consistent with a previous studies that were conducted among adolescents attending secondary schools. For instance, a cross sectional study by Ali et al. that investigated sexual and reproductive health knowledge, attitudes and practices among adolescents in rural Thatta, Pakistan, which reported no significant differences between male and female adolescents, indicating universally inadequate knowledge levels among participants in this age group (26). While the overall knowledge levels were similar, gender-specific needs may differ. For example, girls require earlier education about menstruation and menstrual hygiene management (27, 28), while boys may need targeted education about consent, respectful relationships, and understanding female reproductive health to promote supportive behaviors.

### Limitations

The study has several limitations, including the convenience sampling approach, which may introduce selection bias, as participants were students who attended educational sessions and may differ systematically from those who did not attend. In addition, the current study allows the identification of associations, but does not identify the causal relationships between information sources, barriers, and reproductive health knowledge outcomes. Another drawback, is that the data were collected only in one city, Astana. Although the city is large, urban, and ethnically diverse, its social environment, access to digital resources, and educational infrastructure differ from those of other regions. As a result, the findings may not be fully generalizable to adolescents in rural areas, smaller cities, or other regions with different socioeconomic contexts. An additional limitation is the reliance on self-reported data, which may be subject to recall bias or social desirability bias, particularly given the sensitivity of sexual and reproductive health topics. Importantly, the knowledge gains observed in this study were assessed immediately after a single 90-minute educational session. Therefore, this reflect short-term acquisition rather than long-term retention. While the significant improvements indicate that the intervention was effective in enhancing immediate understanding, they may partly represent short-term memorization effects. Thus, future research should incorporate longitudinal designs with delayed post- tests period to examine the retention of knowledge and whether it translates into behavioral change.

## Conclusion

Several barriers continue to limit adolescents’ access to reproductive health information in Kazakhstan, including parental reluctance to discuss sexual matters and the insufficient scope of school-based education. This study enhances our understanding of existing knowledge gaps and offers a detailed quantitative assessment of specific domains of reproductive health literacy. The findings demonstrate that these barriers translate into clear, measurable deficits across multiple aspects of reproductive and sexual health knowledge. These insights can serve as an important foundation for designing targeted health promotion strategies and guiding future interventions aimed at improving adolescent reproductive health.

## Data Availability

The original contributions presented in the study are included in the article/Supplementary Material, further inquiries can be directed to the corresponding author.
